# Determinants of caregiver satisfaction with child neurodevelopmental assessment in neuropaediatric clinics

**DOI:** 10.1186/s12913-021-06153-5

**Published:** 2021-02-12

**Authors:** Katarina Smejda Kjærandsen, Per Håkan Brøndbo, Marianne Berg Halvorsen

**Affiliations:** 1 RKBU Nord, Faculty of Health Sciences, UiT Arctic University of Norway, Tromsø, Norway; 2 Department of Paediatric Rehabilitation, University Hospital of North Norway, Tromsø, Norway

**Keywords:** Neuropaediatric, Neurodevelopmental assessment, Caregiver satisfaction, Caregiver experiences, Health care services research, Health care surveys

## Abstract

**Background:**

In addition to patient evaluations, caregiver evaluations and experiences are important indicators of the quality of health services. The aim of this study was to examine determinants of caregiver satisfaction with and perceived benefit of child neurodevelopmental assessment in neuropaediatric clinics.

**Methods:**

The study was conducted among caregivers of children and adolescents aged 4–18 years (*N* = 330) referred for neurodevelopmental assessment in two neuropaediatric clinics in the specialised health service in Northern Norway. The Generic Short Patient Experiences Questionnaire (GS-PEQ) for child psychiatric outpatient patients was distributed to caregivers immediately following the assessment, and two of its items were used as measurements of caregiver satisfaction with and perceived benefit of the assessment.

**Results:**

Caregiver satisfaction with the assessment was correlated with a better general level of function in the child, higher socioeconomic status, Norwegian mother tongue, referral from a specialist, and the respondent being a woman. Higher perceived benefit of the assessment was correlated with higher socioeconomic status, Norwegian mother tongue, and younger age of the child. Regression analysis revealed that caregivers’ perception that the assessment was suited to their child’s situation and that there was good cooperation with other public services (e.g., primary care and social/educational services) seemed more fundamental to caregiver satisfaction with neuropaediatric clinics’ services than any background variable. Younger age of the child, in addition to caregivers’ perception that the assessment was suited to their child and receiving sufficient information about the child’s diagnosis/afflictions, were essential to the perceived benefit of the assessment.

**Conclusions:**

Caregiver satisfaction with child neurodevelopmental assessment in neuropaediatric clinics partly depends on variables not related to the assessment experience per se. An assessment that was suited to the child, good cooperation with other public services such as primary health care and social/educational services, and giving sufficient information about the child’s diagnosis are essential to an overall positive caregiver evaluation of neurodevelopmental assessments.

## Background

User experiences with health services can be viewed as reports on the quality of these services [1]. Indeed, patient experiences and satisfaction are associated with important quality aspects of health care, like patient adherence to treatment, patient safety, and clinical effectiveness [2–4]. Information on caregiver experiences with neuropaediatric health services or services for disabled children are increasingly sought [5–8] and are important indicators of the quality of health care delivered to children.

The concepts ‘experiences with health care’ and ‘satisfaction’ are positively related [9]. Measuring experiences with health services gives providers the opportunity to improve care, meet patients’ expectations, and effectively manage and monitor health care performance [9, 10]. Patient satisfaction is a complex concept that depends on several variables, such as social standards, context, needs, values, previous experiences, future expectations, information, education, health, medical care, treatment, and psychological factors [2, 11–13]. Satisfaction surveys are used to capture patient evaluations of many different services and are implicitly or explicitly based on the understanding of satisfaction as the fulfilment of expectations [14]. Reported high satisfaction does not necessarily indicate a good experience, and reported dissatisfaction may be used as an indicator of some negative experience [14]. A study by Norman and colleagues [15] reported that, even when treatment outcomes were poor, satisfaction with Child and Adolescent Mental Health Services (CAMHS) was still high. Collecting information about specific experiences with concrete aspects of health services is a more valid measure of satisfaction, and easier to interpret than satisfaction ratings [16]. Both user experiences and user satisfaction are increasingly employed as quality indicators in the health care sector [17, 18].

From a user perspective, the main components of the quality of service relate to access to information, respect, support, and good coordination and collaboration [2, 19–24]. Parents with children receiving a first-time diagnosis of developmental disability were more satisfied when a large amount of information was provided, and especially appreciated it when clinicians possessed good communication skills and had an understanding of their situation [25]. Previously reported user satisfaction following neuropsychological evaluation [8] was mostly related to clinicians’ concern and competence.

In a study of user satisfaction following paediatric neuropsychological evaluation, Bodin et al. [6] concluded that neither wait time nor referral source were associated with this variable. Holmboe and colleagues [24] found that the perceived wait time for a consultation was associated with parents’ experiences with mental health services, but found no association with the wait time recorded in patient journals. Other results indicated that patient satisfaction with child outpatient services may be related to shorter recorded wait times [26, 27].

Some demographic factors have been repeatedly related to user satisfaction and may be treated as proxies of expectations. Earlier studies have indicated that more positive parental evaluation of health services was related to younger children [19, 21–23, 28–30], and shorter parental education [6, 7, 24, 30–32]. No significant differences in evaluations by caregivers’ mother tongue [22, 24], and in most studies, no significant differences for the gender of the child was found [6, 21, 22, 29, 33]. The reported role of socioeconomic status [2, 34] and the gender of the respondent [18, 35] in satisfaction with health services have been inconsistent.

Generally, good health status of the respondent is associated with a positive evaluation of health services [2, 18, 32, 36, 37]. Parents with poorer health, a lower quality of life, and those who experience more everyday stress may have more negative views of their child’s treatment [38–42]. Therefore, it is important to examine to what extent parents’ mental health can influence their evaluation of neuropaediatric services.

Different results have been reported for the relationship between children’s diagnoses [6, 19, 21, 23, 24, 29, 43] or the number of child health problems and caregiver evaluations of health services [23, 24, 29, 31, 42]. Thus, it is still unclear whether caregivers are experiencing and assessing child rehabilitation services in the context of the severity of their child’s problems. Enhancing knowledge in this area would help clinicians and health services to identify those caregivers who need more information and support, as well as contribute to higher satisfaction with neuropaediatric services. Many earlier studies on user evaluations of health services for disabled children included a limited number of factors that could affect caregiver evaluations; they focused mostly on user experiences with health services and their relationship with demographic variables [6, 19, 21–23, 44].

The aim of the current study was to examine determinants of caregiver satisfaction with and perceived benefit of child neurodevelopmental assessment in neuropediatric clinics in Northern Norway. The outcomes caregiver satisfaction and perceived benefit of assessment were measured by a short-form survey, the Generic Short Patient Experiences Questionnaire (GS-PEQ) [1], to reduce the burden of collecting and analysing data [45]. Given the inconsistent results of other studies, we aimed to investigate the association between background variables (health service characteristics, caregiver characteristics, and child characteristics), as well as specific experiences with neuropaediatric services, and the outcome variables.

## Methods

### Participants

The study population consisted of caregivers of children referred by a general practitioner or a medical specialist to the neuropaediatric outpatient clinics at the University Hospital of North Norway or the Finnmark Hospital Trust in Norway for neurodevelopmental or neurological assessment [46–48]. These clinics are specialised health service units in Northern Norway serving children and adolescents with neurodevelopmental disorders, early-acquired disabilities or intellectual and developmental disabilities. The participants were included consecutively in the present study. In order to be included in the study, children had to be aged 4 to 18 years at the time of referral, and referred between October 2012 and July 2016 at the University Hospital of North Norway, or between January 2014 and July 2016 at the Finnmark Hospital Trust. A total of 518 children and adolescents met these criteria, of whom 153 (30%) were excluded due to lack of treatment in the clinics either because of time constraints, lack of caregiver motivation, or insufficient knowledge of the Norwegian language because several of the questionnaires were only available in Norwegian or a restricted number of additional languages [46]. The caregivers of the remaining 365 eligible children (247 referred by general practitioner, 118 referred by medical specialist) were invited to complete the GS-PEQ and participate in the study. Three hundred thirty caregivers agreed (90.4%) and were included in the final study sample.

The most frequent diagnostic groups among children in the sample were specific developmental disorders (41.5%), intellectual disability (21.8%), autism spectrum disorder (15.8%), and diseases/disorders of the central nervous system such as epilepsy and cerebral palsy (14.8%). The diagnoses were not mutually exclusive. A total of 12% children were not diagnosed with any neurological or neurodevelopmental disorder.

### Measures

#### The generic short patient questionnaire

The GS-PEQ [1], created by The Norwegian Knowledge Centre for the Health Services, is a generic, 10-item, questionnaire that collects information on user experiences across a range of specialist health services. The GS-PEQ is based on previous testing of six, group-specific questionnaires, among them parents’ evaluation of their experiences with somatic inpatient services [49] and psychiatric outpatient services (i.e., CAMHS patients) [50]. The GS-PEQ includes items regarding specific experiences with clinical services, user involvement, information, organisation, accessibility (wait time), incorrect treatment, and outcome (satisfaction and perceived benefit) [1]. The questionnaire’s authors also added three items relevant to CAHMS patients: one regarding clinical services, one regarding information about the assessment, and one regarding cooperation [51]. This version is referred to as the “Generic short version – caregivers about CAMHS” of the GS-PEQ [51], which was used in this study. All 13 items in the applied version of the GS-PEQ are formatted as questions. Twelve of them are answered on a 5-point scale from “not at all” (0) to “to a great extent” [4], or as “not applicable”. One question regarding the wait time to get an appointment is answered on a 4-point scale from “no wait time” (0) to “way too long” [3]. Two of the questions about wait time and incorrect treatment did not correlate or correlated weakly with the other scores in the GS-PEQ, and they were used for comparison with administrative data, respectively in the Parent Assessment of Outpatient CAMHS [50, 52] and the Parent Experiences of Paediatric Care [49]. GS-PEQ is freely available without a license.

#### Background variables

Caregivers’ demographic data (age, gender, marital status, mother tongue, education, and employment) were collected from the appendix that was distributed with the GS-PEQ [51]. Children’s demographic data (age and gender) were taken from the Development and Wellbeing Assessment (DAWBA [53];). Information about referral source (general practitioner or medical specialist) and wait time for the assessment was taken from patient records. A single subscale of the Family Stress Scale part of the DAWBA, socioeconomic/housing score [53], was employed to assess subjective experience of socioeconomic status in the previous 12 months. The variable consisted of items about subjectively evaluated stressors connected to financial difficulties, unemployment, problems with neighbours/neighbourhood, and having home inadequate for family’s needs. Caregivers rated the items on a 3-point scale from “none, or doesn’t apply” (0) to “a lot” [2]. Scores equal to two or higher were assigned lower socioeconomic status (score = 0). We have license to use the DAWBA including the Everyday Feeling Questionnaire through the Youth in Mind: https://youthinmind.com/

#### Caregivers’ mental health

The mental health of the caregivers was assessed with the self-administered version of the Everyday Feeling Questionnaire (EFQ [54]), which is part of the DAWBA [53]. The EFQ contains 10 items estimating symptoms of distress (e.g. “stressed” or “very unhappy”), and psychological well-being (e.g. “positive about the future” or “calmed and relaxed”). Respondents rated the symptoms on a 4-point scale ranging from “none of the time” (0) to “all of the time” [4]. Lower total scores reflect lower levels of distress and higher levels of well-being. The EFQ has good internal consistency, with a Cronbach’s α reported between .87 and .90 [48, 54, 55].

#### Children’s global assessment scale

The CGAS [56] is a clinician-rated tool used to assess the global psychosocial functioning of children, taking into account all available information. The score on this scale reflects the lowest overall level of psychosocial functioning (i.e., at home, at school, and with peers) of the child or adolescent during the preceding month. Total CGAS score ranges from 1 (the most impaired level) to 100 (the best level of functioning), and the score is separated into 10-point intervals, each of which describes a specific level of functioning, followed by examples of matching behaviour and life situations adequate for children and adolescents. In a large Norwegian study of clinicians in outpatient CAMHS [57], the interrater reliability of the routine use of the CGAS was found to be moderate (intraclass correlation coefficient = .61). We have license to use the CGAS through The Norwegian Directorate of eHealth: https://ehelse.no/english

### Procedure

Children underwent the interdisciplinary assessment of neurodevelopmental/neurological disorders and an additional assessment of the presence of coexisting behavioural and emotional disorders. The interdisciplinary assessment included specialists such as paediatricians, neuropsychologists, special education therapists, occupational therapists, and physiotherapists [46, 47]. The presence of a neurodevelopmental/neurological was examined by paediatricians using methods such as MRI Caput, EEG or genetic testing if indicated. A clinical psychologist/neuropsychologist assessed developmental level in all children using a standardised intelligence scale and the Vineland Adaptive Behaviour Scale II [58]. The GS-PEQ was distributed to and completed by caregivers immediately following child neurodevelopmental assessments. Informed consent was obtained from all individual participants included in the study. The study was approved by the appropriate ethics committee. The use of de-identified data was approved by the data protection officer at University Hospital of North Norway and Finnmark Hospital Trust.

### Statistics

The data were analysed using SPSS Version 25. For categorical variables, dummy variables were used (e.g., gender: 0 – man/boy, 1 – woman/girl). Some dummy variables were created for variables with more categories (e.g., mother tongue: 0 – Norwegian, 1 – others; education: 0 – primary/secondary/high school; 1 – college/university). Sami mother tongue was combined with Norwegian mother tongue, as the three participants declaring Sami mother tongue also reported Norwegian mother tongue.

Missing response on items, ceiling effect, demographic characteristics of the respondents, and specific experiences with neuropaediatric services were assessed using descriptive statistics, based on the total number of participants (*N* = 330). An acceptable ceiling effect is generally defined as a maximum of 50% of respondents choosing the most positive response category [59]. Specific experiences with neuropaediatric services were categorised as positive (the two highest item scores), neutral (moderate item scores), and negative (the two lowest item scores). The question “Do you believe that your child was in any way given the wrong treatment?” was excluded from the analyses due to probable misunderstanding by the participants (high scores for wrong treatment were associated with positive experience and satisfaction, *n* = 15).

The relationships between the outcome variables (satisfaction and perceived benefit of the assessment), service characteristics (clinic site, wait time, referral source), caregiver characteristics (gender, mother tongue, socioeconomic status, education, mental health), and child characteristics (age, gender, psychosocial functioning) were examined using Pearson’s correlation coefficients. Hierarchical linear regression analyses were conducted to examine which variables could uniquely explain variation in the outcome variables. The predictive variables included service characteristics, caregiver characteristics, child characteristics, and specific experiences with neuropaediatric services. In correlation and regression analyses, only cases with data both on satisfaction and perceived benefit were used (*N* = 265) to assure that exactly the same participants were used both to predict satisfaction and perceived benefit. These 265 participants had missing values for the following variables: socioeconomic status (16.6%), caregiver gender (8.3%), CGAS score (3.4%), and specific experience with neuropaediatric services (between 0.4 and 13.4%). Missing values were not substituted and were dealt with in linear regression by pairwise deletion. We assessed the significance of change in explained variation (R^2^) by applying a conventional R^2^ change of 2% as a small effect, a change of 13% as a medium effect, and a change of 26% as a large effect [60]. The statistical methods were set on a 5% significance level.

## Results

### Caregiver characteristics

Of 365 caregivers invited to answer the GS-PEQ-CAMHS, 330 completed it (90.4%). Respondents were between 24 to 71 years of age (mean, *M* = 41.5; standard deviation, *SD* = 7.4). Children’s age ranged between 4 and 18 years (*M* = 10.2, *SD* = 3.8), and 34.2% were females. Detailed caregiver characteristics are included in Table 1. There were no significant differences in service or child or characteristics between caregivers who completed the GS-PEQ and those who did not.

**Table 1:** Demographic characteristics of caregivers (*N* = 330)

	*N*	%
Caregivers		
Mother	232	70.3
Father	69	20.9
Mother and father	10	3.0
Other	19	5.8
Marital status		
Married	163	49.4
Cohabitant	104	31.5
Without partner	63	19.1
Mother tongue		
Norwegian	299	90.6
Other Nordic language	7	2.1
Other European language	16	4.8
Non-European language	8	2.4
Education level of caregiver		
Primary/Secondary School	29	7.9
High School	143	43.6
College/University up to 4 years	104	31.7
University above 4 years	52	15.9
Education level of caregiver’s partner		
Primary/Secondary School	42	14.9
High School	143	50.7
College/University up to 4 years	59	20.9
University above 4 years	38	13.5
Employment status of caregiver		
Gainfully employed	245	74.2
On sick leave, disability pension or rehabilitation	43	13.0
Under education	9	2.7
Working at home	11	3.3
Unemployed	4	1.2
Another activity	18	5.5
Employment status of caregiver’s partner		
Gainfully employed	212	75.4
On sick leave, disability pension or rehabilitation	40	14.2
Under education	11	3.9
Working at home	4	1.4
Unemployed	2	0.7
Another activity	12	4.3

### Ceiling effect, missing values, and not applicable responses

Only one question about perceived benefit of the assessment met the criterion of maximum 50% responses in the most positive category. All the other questions achieved a high ceiling effect. Missing values occurred in around 2% of answers, with a range 0.9–7% (Table 2).

**Table 2:** Caregivers’ specific experiences with neuropaediatric services. Questions from the Generic Short Patient Experiences Questionnaire (GS-PEQ)

Question	Missing (%)	Not applicable (%)	Applicable (*N*)	Ceiling effect (%)	Positive experience (%)	Neutral experience (%)	Negative experience (%)	*M*	*SD*
Did the clinicians talk to you in a way that was easy to understand?	0.9	1.2	318	84.9	99.1	0.6	0.3	3.83	.44
Do you have confidence in the clinicians’ professional competence?	1.2	1.2	317	82.3	99.4	0.6	0.0	3.82	.40
Do you have confidence in the other staff’s professional skills?	1.5	7.9	294	74.5	98.0	2.0	0.0	3.72	.49
Were you told as much as you considered necessary about how tests or examinations would be carried out?	1.2	0.6	319	68.3	92.2	7.2	0.6	3.60	.67
Did you get sufficient information about your child’s diagnosis/afflictions?	4.5	11.8	271	57.6	86.7	10.0	3.3	3.39	.88
Did you perceive the treatment that you child received as suited to his/her situation?	1.8	8.5	291	64.6	94.5	4.8	0.6	3.58	.63
Were you involved in any decisions regarding your child’s treatment?	4.2	22.1	238	56.7	86.1	8.0	5.9	3.34	.94
Did you perceive the clinic’s work as well organised?	1.8	2.1	312	63.5	92.2	6.9	0.9	3.54	.68
Do you find that the clinic cooperated well with other public services?	3.0	20.9	246	50.4	83.7	13.1	3.2	3.30	.82
Overall, was the help and treatment you received at the clinic satisfactory?	3.3	7.3	290	68.6	97.0	2.7	0.3	3.65	.57
Overall, what benefit have you had from the care at the clinic? *	7.0	8.2	280	38.2	83.9	15.0	1.1	3.20	.77

*Note*. “Missing” and “not applicable” based on initial *N* = 330; Ceiling effect (acceptable ceiling effect was defined as a maximum of 50% of respondents choosing the most positive response category), and positive (the two highest item scores), neutral (moderate item scores), and negative experiences (the two lowest item scores) based on *N* applicable; A five-point response scale was used for 10 items: 0 – not at all, 1 – to a small extent, 2 – to a moderate extent, 3 – to a large extent, 4 – to a very large extent, 5 – not applicable; * A five-point response scale with different answers was used to this question: 0 – no benefit, 1 – a small benefit, 2 – a moderate benefit, 3 – a large benefit, 4 – a very large benefit, 5 – not applicable

Caregivers had the possibility to choose “not applicable” in response to all the questions about specific experiences, as well as for the outcome variables. They judged two questions as especially irrelevant to their situation: “Were you involved in any decisions regarding your child’s treatment?” (22.1% answered “not applicable”) and “Do you find that the clinic cooperated well with other public services?” (20.9% answered “not applicable”). Most of the questions that contained the word “treatment” had a high percentage of “not applicable” answers. Caregivers that chose “not applicable” to answer the question on information about diagnosis/afflictions were statistically significantly more likely to have a child without any diagnosis of neurodevelopmental/neurological disorder (χ^2^ = 6.6, *p* = .01). All the questions concerning communication with the clinician and confidence in his/her professional skills were highly applicable (only around 1% “not applicable”), with exception of confidence in the professional skills of other staff (8% responded “not applicable”).

### Caregiver evaluation of the assessment

Most caregivers were highly satisfied with their child’s assessment (97%) (Table 2), and they answered positively to almost all questions about the relationship with clinicians (i.e., communication and confidence in their professional skills). Caregivers evaluated the assessment as highly beneficial (83.9% positive experiences). Most negative experiences were related to caregivers’ involvement in decisions regarding the child’s assessment (5.9%), the information they were given about their child’s diagnosis or afflictions (3.3%), and the clinic’s cooperation with other public services (3.2%).

### Determinants of caregiver satisfaction with and perceived benefit of the neurodevelopmental assessment

Caregiver satisfaction with and perceived benefit of the child neurodevelopmental assessment were moderately correlated (*r* = .47, *p* < .001). Satisfaction with the assessment was weakly associated with referral from a specialist, being a woman, having Norwegian mother tongue, higher socioeconomic status, and having a child with higher psychosocial functioning (Table 3). Perceived benefit of the assessment was weakly related to having Norwegian mother tongue, higher socioeconomic status, and being caregiver to a younger child. Wait time, caregiver’s education and mental health, and child’s gender did not have any significant association with the outcome variables. Caregiver satisfaction with and perceived benefit of the assessment did not differ significantly between the two clinic sites (University Hospital of North Norway and the Finnmark Hospital Trust).

**Table 3:** Bivariate relationships between overall caregiver satisfaction with child neurodevelopmental assessment and background variables

	*N*	*M* (*SD*)/*n*(%)	1	2	3	4	5	6	7	8	9	10	11	12
1 Satisfaction	265	3.65 (.58)												
2 Benefit	265	3.22 (.75)	.47 ^***^											
3 Clinic	265	227 (85.7%)	.01	−.09										
4 Referral source	265	79 (29.8%)	−.16 ^*^	−.03	.08									
5 Wait time, days	265	90.29 (53.13)	−.02	−.05	−.08	−.09								
6 Gender of respondent	243	58 (23.9%)	.13 ^*^	.06	.08	.05	−.06							
7 Mother tongue	265	241 (90.9%)	−.17 ^**^	−.13 ^*^	−.09	.06	.12 ^*^	−.15 ^*^						
8 Socioeconomic status	221	21 (9.5%)	.23 ^***^	.14 ^*^	.09	−.01	.06	.02	−.08					
9 Education	265	139 (52.5%)	.05	.04	.04	−.06	.00	.14 ^*^	.04	.05				
10 Mental health	220	11.79 (5.11)	−.11	−.10	.00	.07	.02	.03	.00	−.22 ^***^	−.09			
11 Age of child	265	10.22 (3.88)	−.08	−.20 ^**^	.03	−.03	−.04	.03	−.04	.13	−.01	−.03		
12 Gender of child	265	162 (61.1%)	.01	.02	.09	−.14 ^*^	.01	.04	−.14 ^*^	.14 ^*^	−.06	−.04	.11	
13 Child’s psychosocial functioning	256	56.40 (13.93)	.14 ^*^	.04	.06	−.04	−.09	−.01	.04	.10	.16 ^**^	−.12	−.02	.12

*Note. N* including only those who answered both questions about satisfaction and perceived benefit with the assessment. Clinic: 0 – University Hospital of North Norway, 1 – Finnmark Hospital Trust; referral source: 0 – medical specialist, 1 – general practitioner; gender: 0 – male, 1 – female; mother tongue: 0 – Norwegian, 1 – others; socioeconomic status: 0 – lower, 1 – higher; education: 0 – lower, 1 – higher; mental health: a score from the Everyday Feeling Questionnaire, higher scores mean higher distress in a caregiver; child’s psychosocial functioning measured by CGAS (Children’s Global Assessment Scale); *p < .05. **p < .01, ***p < .001 (two-tailed test)

The overall model predicting caregiver satisfaction with the neurodevelopmental assessment was significant (*F*(15,158) = 13.03, *p* < .001) and accounted for 55.3% of the variance in the satisfaction score (Table 4). Background variables (step 1) and specific experiences with neuropaediatric services (step 2) accounted for 13.7, and 41.6% of the variance in satisfaction, respectively, reflecting an effect of medium magnitude in step 1, and an effect of large magnitude in step 2. Specifically, satisfaction was significantly associated with two kinds of specific experiences with neuropaediatric services: perceived suitable assessment and cooperation with other public services (i.e., primary care and social and educational services).

**Table 4:** Hierarchical multiple regression analysis results for the prediction of caregiver satisfaction with and benefit of the assessment

Predicting variables	Satisfaction		Benefit	
	Δ*R*^*2*^	β	Δ*R*^*2*^	β
**Step 1: Background variables**	.137***		.084*	
Referral source		−.10		.01
Caregiver’s gender		.04		−.02
Mother tongue		−.02		−.03
Socioeconomic status		.10		.05
Child’s age		−.05		−.20**
Child’s psychosocial functioning		.07		−.04
**Step 2: Specific experiences with neuropaediatric services**	.416***		.227***	
The clinicians easy to understand		.02		−.04
Confidence in the clinicians’ professional competence		.09		−.05
Confidence in the other staff’s professional skills		−.02		.08
Informed about how tests or examinations would be carried out		−.07		.01
Got sufficient information about the child’s diagnosis/afflictions		.05		.17*
The treatment suited to the child’s situation		.48***		.26**
Involvement in any decisions regarding the child’s treatment		.03		.00
Perceiving the clinic’s work as well organised		.07		.14
The clinic cooperated well with other public services		.21**		.10
Total *R*^*2*^		.553***		.310***

*Note.* All β (standardised coefficients) were from the final model with all steps included. Referral source: 0 – specialist, 1 – general practitioner; gender: 0 – male, 1 – female; mother tongue: 0 – Norwegian, 1 – others; socioeconomic status: 0 – lower, 1 – higher; child’s psychosocial functioning measured by CGAS (Children’s Global Assessment Scale); *p < .05. **p < .01, ***p < .001 (two-tailed test)

The overall model predicting the perceived benefit of the assessment was significant as well (*F*(15,158) = 4.74, *p* < .001) and accounted for 31% of the variance in the way caregivers answered to the question about the benefit of the assessment. Background variables (step 1) and specific experiences with neuropaediatric services (step 2) accounted for 8.4, and 22.7% of the variance in benefit, respectively, reflecting an effect of small magnitude in step 1, and an effect of medium magnitude in step 2. Specifically, child’s lower age, caregiver’s perception of a suitable assessment, and getting sufficient information about the child’s diagnosis/afflictions significantly predicted the perceived benefit of the assessment.

## Discussion

The overall purpose of this study was to examine determinants of caregiver satisfaction with and perceived benefit of the child neurodevelopmental assessment. We looked at specific experiences with neuropaediatric services as well. In general, most of the caregivers were satisfied with their child’s assessment in the two neuropaediatric clinics in Northern Norway; similar results have been reported in similar patient populations [6, 8, 29]. In addition, good cooperation with other public services and the assessment suited to the child’s situation seemed more fundamental to caregiver satisfaction with neuropaediatric clinics’ services than any background variable.

As user surveys tend to be positively skewed [13, 14], it was important to look at the few respondents who were not fully satisfied. A relatively high number of caregivers evaluated their involvement in the assessment, the cooperation with other services, and the provision of sufficient information about their child’s diagnosis/afflictions as either more negative or not relevant for them in relation to other specific experiences. Other studies on services for disabled children pointed out that caregivers gave the most negative evaluations for the amount of information received [19, 21–25] and the coordination of delivered services [21], which is in line with our study.

Most of the background variables were negligible in predicting caregiver satisfaction with and perceived benefit of the assessment, especially after specific experiences with neuropaediatric services were included in the regression analyses. These specific experiences explained more of the high overall satisfaction with the assessment than any other background variable. In our study, these specific experiences played a causal role in caregiver satisfaction; and a generic survey that includes single questions on specific indices of different experiences instead of full scales is a good method to identify predictive variables [61]. User experiences with health services were the most powerful determinants of patient overall satisfaction in other studies as well [9, 18, 34, 36]. In our study, two types of specific experiences with neuropaediatric services were especially crucial in the explanation of overall satisfaction, i.e., if the assessment was suited to the child’s situation, and cooperation with other public services, demonstrating that these factors are of high importance. The situation seemed different for the perceived benefit of the assessment. Specific experiences with neuropaediatric services explained this outcome to a smaller degree, and among the background variables, child’s age was still essential to the explanation of the variance in this outcome. Specific experiences that were crucial to the perceived benefit of the assessment were whether the assessment was suited to the child’s situation, and getting sufficient information about child’s diagnosis/affliction after the assessment.

Some background variables were clearly only weakly correlated with caregiver satisfaction and perceived benefit. Caregivers of children referred by general practitioners were less satisfied with the assessment than those of children referred by a medical specialist. This result could be due to the different health problems that may be present in patients referred from a medical specialist, as it is likely that these patients spent more time in specialist health services, and/or had more serious health problems, making the neurodevelopmental assessment an important step in the process of clarifying the child’s afflictions. Caregivers with Norwegian mother tongue were more satisfied with and perceived a higher benefit of the assessment. This difference could be caused by either communication problems or different expectations of health services related to cultural background. Higher socio-economic status was related to both higher satisfaction with the assessment and more perceived benefit of the assessment. Previous results on the relationship between socioeconomic factors and user satisfaction have been inconsistent [2, 34]. In a review, Willems and colleagues [62] concluded that patients from lower social classes could be disadvantaged due to a misperception of their needs on the part of their doctor, as well as their lower ability to participate in the care process. They pointed out that the communication between doctors and these patients was characterised by less information, fewer directions, and less socio-emotional and partnership building. Both our results and the results from the review indicate that clinicians should be aware of contextual differences in their communication patterns with patients/their caregivers. Finally, in our study, caregivers of younger children had a higher perceived benefit of the assessment, confirming other findings [19, 21–23, 28–30]. Younger children are new in the system, and an assessment can be a milestone in understanding the child and learning more about a condition. Another possible explanation is that older children may have more severe neurodevelopmental problems and a higher incidence of mental health difficulties [63]. Egilson [28] explained such results simply by assuming that parents become more critical of the services as their children grow older. Generally, the existence of small associations between demographics and service evaluations can have two explanations – different groups may have different response tendencies or different groups may be treated differently during the care process [12].

Higher child global psychosocial functioning was associated with higher caregiver satisfaction, and this should be taken into consideration when interpreting user satisfaction surveys. This result could indicate that the caregivers of these children needed less help. A study by Ezpeleta et al. [64] showed that parents of children with high functional impairment both more often admitted needing psychiatric help and more often sought such help. It is also possible that expectations of health care delivery did matter [2, 9, 65]. Ambiguous results about the severity of a child’s mental health and service evaluation exist in the literature [6, 19, 21, 23, 24, 29, 31, 43], but our results are in accordance with results that showed an association between higher caregiver satisfaction with services and better functioning [42] or less severe problems of the child [64]. Our finding of no significant relationship between caregiver’s mental health and satisfaction with the assessment disproves earlier findings of an association between the health status of a respondent and service evaluation [2, 18, 32, 36, 37, 40–42].

When the GS-PEQ was created, it was assumed that the number of questions inapplicable to any respondent would not exceed 20% [1]. In our study, as many as one-fifth of the caregivers evaluated questions about involvement and cooperation as inapplicable to their child’s situation. This may indicate that the caregivers did not recognise these areas as a responsibility of the clinics. Such an evaluation of the service could be influenced by the temporal characteristics of the assessment. A cooperative feedback meeting, where the results are communicated and clinical implications and further treatment is planned, takes place within 2 weeks of the assessment. Visible cooperation with other public services, like primary care and social/educational services, also starts then, whereas our caregivers completed their evaluation of health care delivery directly after the assessment. If our caregivers had completed the GS-PEQ after the cooperative feedback meeting, it may have led to a different evaluation.

### Clinical implications

The evaluation of cooperation with other services as inapplicable by many of our caregivers could mean that they did not get clear information about the possibility for cooperation between neuropaediatric clinics and primary care and social/educational services, among others. At the same time, it is difficult to imagine that an assessment in a neuropaediatric clinic could be conducted in a vacuum, without any interaction with other important services. Thus, caregiver evaluations might indicate that The Coordination Reform, which was enacted in Norwegian health care system in the 2000s, and concerned cooperation and coordination across health care units [66], did not affect neuropaediatric services to the extent necessary.

Norwegian national guidelines for child neuropaediatric clinics emphasise the importance of user involvement as a prerequisite for patient and user safety, and a requirement for sound services [67]. Many caregivers in our study replied that the question on being involved in the assessment was inapplicable to their situation. We concluded that the involvement rates and knowledge of the possibility for involvement are definitely areas that require improvement in the clinics. In addition, we found that the use of the term “treatment” in the questionnaire might be problematic, as it could cause respondents to misunderstand the questions (among them the question about involvement); indeed, the health service delivered was primarily an assessment, not a treatment.

### Strengths and limitations

Our study has some significant strengths. There are advantages to our close-to-real-time data collection [68]. We had a very good response rate, and the timing of our data collection prevented memory distortion in the participants. A generic survey has its advantages – it is time-saving, more motivating to complete, creates less burden on participants, is easier to interpret, and allows comparisons between different health care units [69]. Of course, our study has some limitations as well. The GS-PEQ is a survey that was created based on health service-specific surveys; it was meant to cover both adults and children, inpatients and outpatients, and short and long-lasting treatment. However, only two of these surveys refer specifically to children [49, 50], and one of them to outpatients [50]. None of these surveys are specific to child rehabilitation or neuropaediatric clinics. This could create a problem with applicability or suitability of the selected questions, and may have influenced the acceptance or understanding of these surveys by the users. The creators of the GS-PEQ [1] recommended it for the use in large samples to help strategic managers monitor quality of care, and to inform decision-making or service evaluation at the operational management level. In addition, our results were positively skewed, indicating the existence of a ceiling effect. Thus, interpretation of satisfaction can be problematic as the outcome of an active evaluation [14]. Another limitation is that we cannot exclude the possibility that the least satisfied caregivers refused participating in our study, and their lack of participation could influence the results.

## Conclusions

The GS-PEQ contains questions related to a wide spectrum of specific experiences that explained significant proportions of the variation in satisfaction and perceived benefit of the assessment in our study. These specific experiences are indices of the perceived quality of health services. Caregiver satisfaction with neurodevelopmental assessment in neuropaediatric clinics in our study depended partly on variables not related to specific experiences with neuropediatric services per se. However, an assessment that was adapted to the child’s needs, good cooperation with other public services such as primary care and social/educational services, and giving sufficient information about the child’s diagnosis are experiences that are essential to an overall positive evaluation of child neurodevelopmental assessment. In addition, clinicians should be especially vigilant in including caregivers in decision-making and in discussing the possibilities for cooperation with other services.

## Data Availability

The datasets analysed during the current study are not publicly available due to ethical restrictions and personal data protection but are available from the authors on reasonable request and with permission of the Data Protection Official in the health trusts.

## Abbreviations

**CAMHS**: the Child and Adolescent Mental Health Services
**CGAS**: Child Global Assessment Scale
**DAWBA**: Development and Well-being Assessment
**EFQ**: the Everyday Feeling Questionnaire
**F**: ratio of the mean regression sum of squares divided by the mean error sum of squares
**GS-PEQ**: the Generic Short Patient Experiences Questionnaire
**M**: Mean
**N**: total sample size
**n**: subsample size
**p**: probability of the data arising by chance
**r**: Pearson’s product moment correlation coefficient
**SD**: Standard Deviation
**SDQ**: the Strengths and Difficulties Questionnaire
